# Supplementary material for: Phonon transition across an isotopic interface
Source: Nat Commun. 2023 Apr 25;14:2382. doi: 10.1038/s41467-023-38053-z (PMC10130007; doi:10.1038/s41467-023-38053-z)
Supplement: Supplementary file 1 — Supplementary Information [file 41467_2023_38053_MOESM1_ESM.pdf]

Supplementary information for

Phonon transition across an isotopic interface

Ning Li[1, 2, 3#], Ruochen Shi[1, 2#], Yifei Li[4#] , Ruishi Qi[1, 2, 5], Fachen Liu[1, 2, 3], Xiaowen Zhang[1, 2], Zhetong Liu[1, 2, 3], Yuehui Li[1, 2], Xiangdong Guo[6], Kaihui Liu[7, 8], Ying Jiang[1, 8, 9], Xin-Zheng Li[7, 9, 10], Ji Chen[7, 8, 9*], Lei Liu[4, 9*], En-Ge Wang[1, 9, 11, 12*], and Peng Gao[1, 2, 8, 9, 13*]

[1]International Center for Quantum Materials, School of Physics, Peking University, Beijing 100871, China.

[2]Electron Microscopy Laboratory, School of Physics, Peking University, Beijing 100871, China.

[3]Academy for Advanced Interdisciplinary Studies, Peking University, Beijing 100871, China.

[4]School of Materials Science and Engineering, Peking University, Beijing 100871, China.

[5]Physics Department, University of California at Berkeley, Berkeley, CA 94720, USA

[6]CAS Key Laboratory of Nanophotonic Materials and Devices, CAS Key Laboratory of Standardization and Measurement for Nanotechnology, CAS Center for Excellence in Nanoscience, National Center for Nanoscience and Technology, Beijing 100190, China.

[7]Institute of Condensed Matter and Material Physics, Frontiers Science Center for Nano-optoelectronics, School of Physics, Peking University, Beijing 100871, China.

[8]Collaborative Innovation Center of Quantum Matter, Beijing 100871, China.

[9]Interdisciplinary Institute of Light-Element Quantum Materials and Research Center for Light-Element Advanced Materials, Peking University, Beijing 100871, China.

[10]State Key Laboratory for Artificial Microstructure and Mesoscopic Physics, Peking University, Beijing 100871, China.

[11]Songshan Lake Materials Laboratory, Dongguan 523808, China.

[12]School of Physics, Shanghai University, Shanghai 200444, China.

[13]Hefei National Laboratory, Hefei 230088, China

[#]These authors contributed equally: Ning Li, Ruochen Shi, Yifei Li

**Corresponding author. Email: ji.chen@pku.edu.cn, l_liu@pku.edu.cn, egwang@pku.edu.cn, p-gao@pku.edu.cn*

Supplementary note 1：First-principles calculations

Structure and phonon calculations. Density functional theory (DFT) calculations of the structure and phonon properties of h-BN bulk and isotopic heterojunction were performed using Quantum ESPRESSO [1,2] the Vanderbilt ultrasoft pseudopotentials [3]. The local density approximation (LDA) with the Perdew-Zunger parameterization [4] and the generalized gradient approximation with the Perdew-Burke-Ernzerhof [5] were considered for the exchange-correlation functional. The kinetic energy cut-off was 50 Rydbergs (Ry) for wavefunctions and 500 Ry for charge density and potential. A 9 × 9 × 3 k-points mesh was used for bulk hBN, while a 6 × 6 × 1 k-points mesh was used for the heterojunction. The heterojunction model contains 10 unit-cells of h-^{10}BN and 10 unit cells of h-^{11}BN connected along z direction (80 atoms in one hexagonal unit cell). The structure was optimized until the residual force was below 10^{-4} Ry per Bohr on every atom. The dynamic matrices and force constants were obtained using density functional perturbation theory (DFPT). The spatially resolved EELS cross-section calculation in Fig. 2b was performed following the same formula as literature [6]. The phonon dispersion and projected phonon density of states in supplemental figure 9 is obtained using DFPT from the bulk hBN and Graphite.

Phonon induced differential charge density calculation. To calculate momentum- and mode-resolved phonon-induced differential charge density, we first interpolate the dynamic matrix of heterojunction on a 10 × 10 × 1 q-mesh, and diagonalize it to get the eigenvector $\xi_{i,\alpha,\lambda,q}$, representing the eigenvector of ith atom, λth mode at q point in Cartesian coordinate direction α. Then the atomic displacements are generated based on $d_{i,\alpha,\lambda,q} = \xi_{i,\alpha,\lambda,q}/\sqrt{m_i}$, where $d_{i,\alpha,\lambda,q}$ is the displacement and m_i is the mass of ith atom. The charge density of each displaced configuration is then calculated using DFT with the same parameter as described before. The thermal excitation coefficients under finite temperature are calculated by molecular dynamics using the LAMMPS package [7] with the Tersoff interatomic potential parametrized in ref. [8]. The

displacement of ith atom $x_{i,\alpha}$ can be written as $x_{i,\alpha}(t) = \sum_{\lambda,q} \xi_{i,\alpha,\lambda,q} \cdot \frac{1}{\sqrt{m_i}} \cdot e^{-i\omega_{\lambda,q}t} \cdot C_{\lambda,q}$, where $\omega_{\lambda,q}$ is the frequency of λth mode at q point, $C_{\lambda,q}$ is the thermal excitation coefficient. Using the fact $\sum_{i,\alpha} \xi_{i,\alpha,\lambda,q}{}^* \cdot \xi_{i,\alpha,\lambda,q} = 1$, we can get $C_{\lambda,q} = \sum_{i,\alpha} \xi_{i,\alpha,\lambda,q}{}^* \cdot \sqrt{m_i} \cdot X_{i,\alpha,\lambda,q}$, where $X_{i,\alpha,\lambda,q}$ is the λ, q component of the Fourier transform of $x_{i,\alpha}(t)$. The charge density of low-q modes and high-q modes are averaged with weight $C_{\lambda,q}$ respectively, where low-q modes are defined as its distance to Γ point is smaller than the half of the side length of Brillouin zone, and the other modes are defined as high-q modes. The differential charge density is then obtained by the difference between the averaged charge density under phonon perturbation and the charge density of unperturbed structure. For each configuration, the differential charge density of perturbation along the positive and negative, i.e., +z direction and -z direction, is averaged. The interfacial charge is calculated as the integral of the differential charge density over one adjacent unit cell (~0.34 nm) of the interface each side. The line profile of differential charge density is convoluted with a gaussian factor considering the limited beam size.

Supplementary note 2: Influence of the SiO_2 signals on h-BN isotopic phonon analysis

As shown in the Supplemental Figure 3b below, the peak at ~130 meV is the phonon polaritons of SiO_2, which can be detected in vacuum under aloof mode [9]. The energy window of this signal has no overlap with h-BN, i.e., 160-200 meV for in-plane and 80-100 meV for out-of-plane. Thus, it would not affect the analysis of h-BN phonons. The peak at ~100 meV is predominantly excited by impact scattering [10], which decays quickly with no energy variation [9]. Therefore, the very short-range interaction of this signal makes little influence to our analysis on the out-of-plane phonons of h-BN.

Supplementary note 3: Influences of the tilt angle on the vibration signals

As shown in the Supplemental Figure 4 below, we built h-BN cross-sectional

structures with different crystal axis directions, including [1 0 0], [10 1 0], [5 1 0], [10 3 0], [5 2 0] and [2 1 0]. The corresponding tilt angles are 0°, 5.74°, 11.54°, 17.46°, 23.58° and 30°, respectively. These typical tilt angles can basically reflect the trend of the influence on the phonon signals variation in EELS. The EELS vibrational spectrum calculated by DFPT is shown in Fig. S4b, where colored arrows highlight the dominant vibration modes in each spectrum. In order to study the influence of the zone-axis deflection on vibrational spectrum, we quantitatively extracted peak position and signal intensity of the main vibration modes. Fig. S4c shows the change of the energy of each mode as the tilt angle increases, and Fig. S4d shows the change of the intensity of each mode with the tilt angle increases.

When the tilt angle reaches the maximum 30°, the energy of TO mode has a blue shift up to ~10 meV, and its intensity changes by more than 2 times (as seen in the middle panels of Fig. S4c, d). However, when the tilt angle is small, the energy shift is not significant. For example, with 5° tilt, the energy shift of TO mode is less than 0.2 meV, which is even small than the typical precision of EELS thus can be neglected during the quantitative analysis for the in-plane vibrational signals, while for the ZO modes along out-of-plane direction, the energy shift is smaller than 1.1 meV. So the tilt angle between the heterostructure needs to be controlled to a small extent in the experiments. In addition, we find that the energy changes of in-plane modes are non-linear, while the out-of-plane modes show linear relationship to the tilt angle.

In fact, in our experiments the sample have always been rotated to make the electron beam incident at the middle angle of two isotope flakes. Under this circumstance, the tilt angle between sample and electron beam is halved. Therefore, the two parts have the same tilt angle thus do not affect the comparison of signals between the two parts. For example, for data presented in the manuscript, the angle between the h-^{10}BN/h-^{11}BN isotopes is experimentally measured to be ~10° by rotating the h-^{10}BN h-^{11}BN part successively to zone axis with the assistance of convergent-beam electron diffraction. The sample has been rotated to the middle of h-^{10}BN/h-^{11}BN layer, i.e., the angles between the electron beam and the h-^{10}BN /h-^{11}BN layer are both ~5°. In this

case, we estimate the angle deviation between these two flakes should be smaller than ~5°, for which the angle effect can be completely negligible compared the instrumental precision.

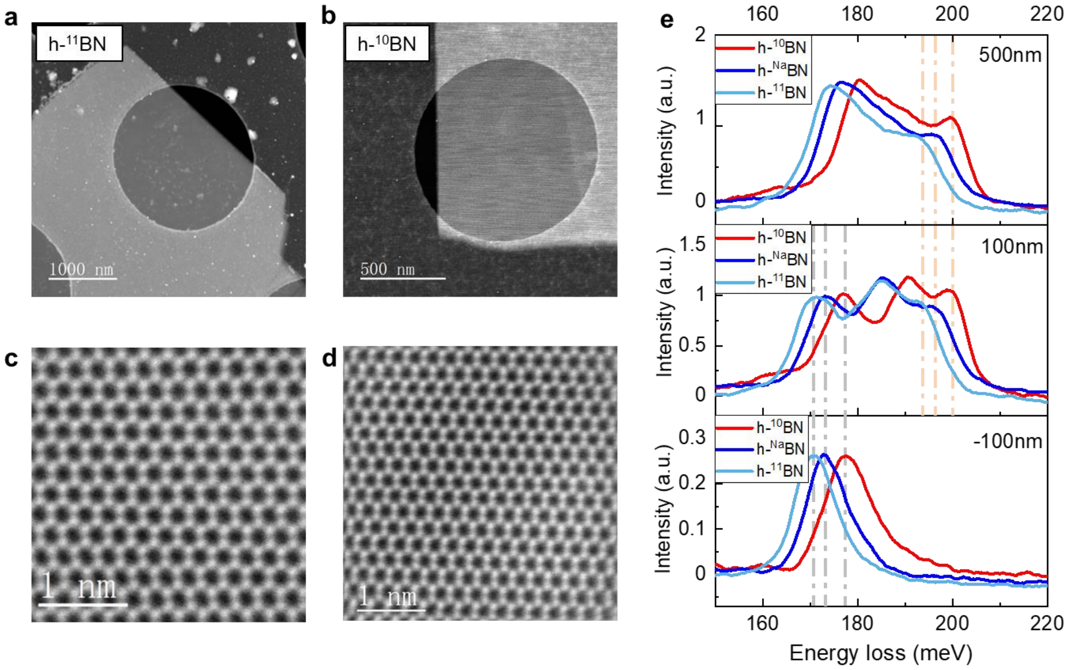

Supplementary figure 1 | Isotope enriched hBN flakes. (a, b) Low magnification HAADF image of h-^{11}BN and h-^{10}BN crystals. **(c, d)** Atomically resolved HAADF image of multilayer h-^{11}BN and h-^{10}BN, showing high quality. The image has been denoised using Gaussian blur. **(e)** Vibrational STEM-EELS spectra of h-^{10}BN, h-^{Na}BN and h-^{11}BN at different positions, presenting the good isotopic separation ability of EELS. Negative distance corresponds to the aloof mode acquisition. The dashed vertical grey lines denoting the signals of phonon polariton, orange lines denoting the signals of LO phonon modes of h-BN.

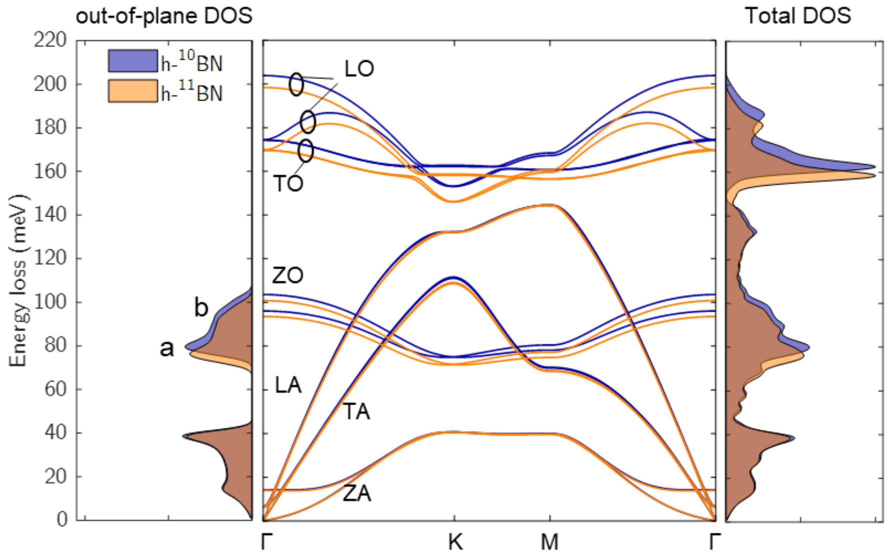

Supplementary figure 2 | DFT calculated phonon dispersion of h-^{10}BN and h-^{11}BN isotopes, and corresponding phonon density of states (DOS). The left part is the DOS of out-of-plane component and the right part is the total DOS of all directions. At the range of 80-100 meV there are two dominate peaks a and b, which are the out-of-plane optical phonons (ZO). Peaks a and b are originated from phonons with different q, thus we labeled them as $ZO_{high\ q}$ (at BZ bounary) and $ZO_{low\ q}$ (at BZ center) in the manuscript.

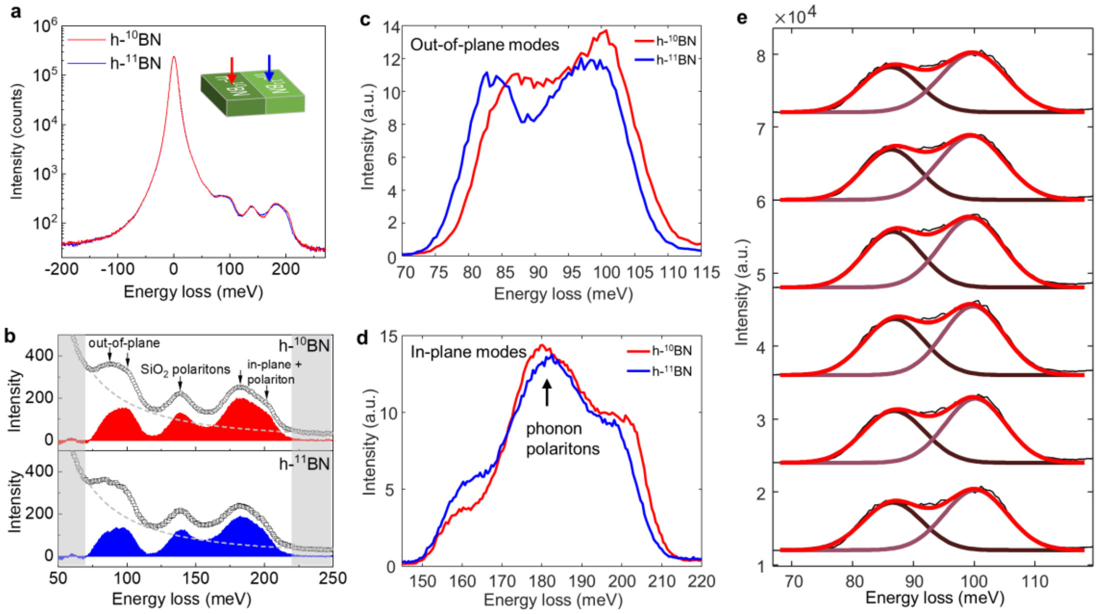

Supplementary figure 3 | EELS processing and the fitting of ZO modes. (a) Original EELS spectra acquired at h-^{10}BN region and h-^{11}BN region in log scale. **(b)** The dots are summed raw EELS data, dashed lines are the fitted background using the modified Pearson-VII function (fitting windows are 55-70 meV and 220-250 meV), the filled red and blue curves are the background-subtracted signals. **(c, d)** The background-subtracted signals of out-of-plane and in-plane modes de-convoluted using Lucy-Richardson algorithm. The in-plane modes are significantly influenced by the phonon polaritons of h-BN, of which the signal energy is sensitive to the edge distance and sample geometry. **(e)** Multi-gaussian fitting of the out-of-plane signals. These spectra are acquired at different positions between two atomic layers.

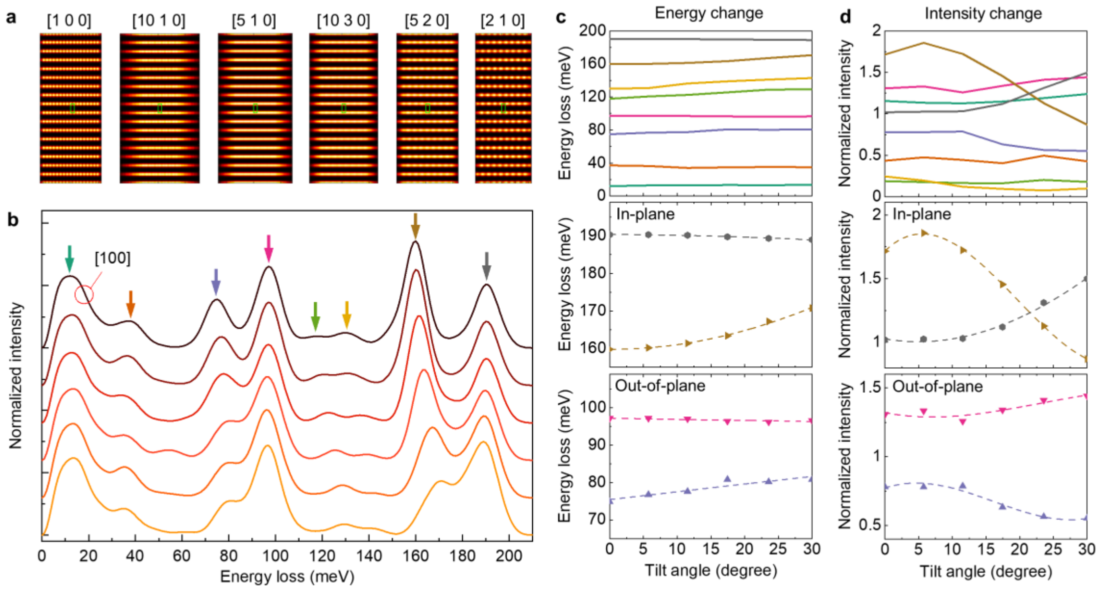

Supplementary figure 4 | Influence of sample tilt on the vibrational spectrum measurement. (a) Schematic diagram of the cross-section h-BN at different tilt angles. **(b)** EELS vibrational spectra at different tilt angles calculated by DFPT. **(c)** The relationship between tilt angle and the energy of different vibrational modes. The in-plane modes and out-of-plane modes are highlighted in the middle and the bottom panels, respectively. With tilt angle at 30°, the maximum energy change is ~10 meV for the in-plane modes, and ~5meV for the out-of-plane modes. In addition, the energy changes of in-plane modes are non-linear, while the out-of-plane modes show linear relationship to the tilt angle. **(d)** The relationship between tilt angle and the intensity of different vibrational modes. The in-plane modes and out-of-plane modes are highlighted in the middle and the bottom panels, respectively.

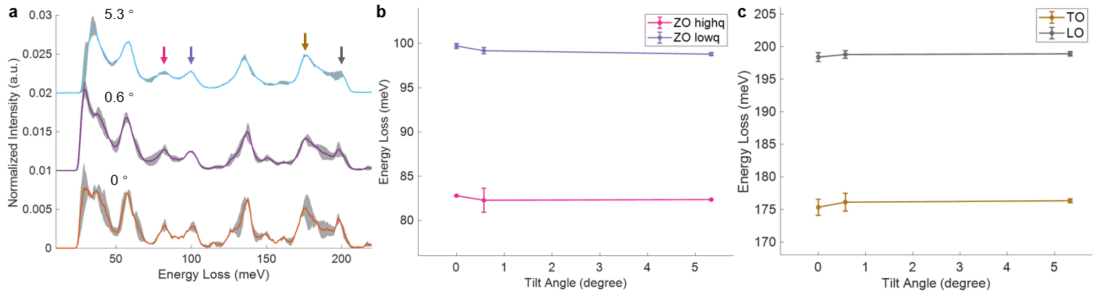

Supplementary figure 5 | The influence of tilt angle on the EELS spectra of h-BN. **(a)** The EELS spectra of $h^{10}BN$ with tilt angle of 0° (orange), 0.6° (purple) and 5.3° (cyan). The gray shades are the standard deviations. **(b)** the energy of ZO high-q (magenta) and ZO low-q (blue) modes with tilt angle. **(c)** the energy of LO (gray) and TO (yellow) modes with tilt angle. Error bars are standard deviations. The energy changes are within 1 meV in experimental tilt angle range, much lower than the energy change caused by the isotope effect (~3 meV).

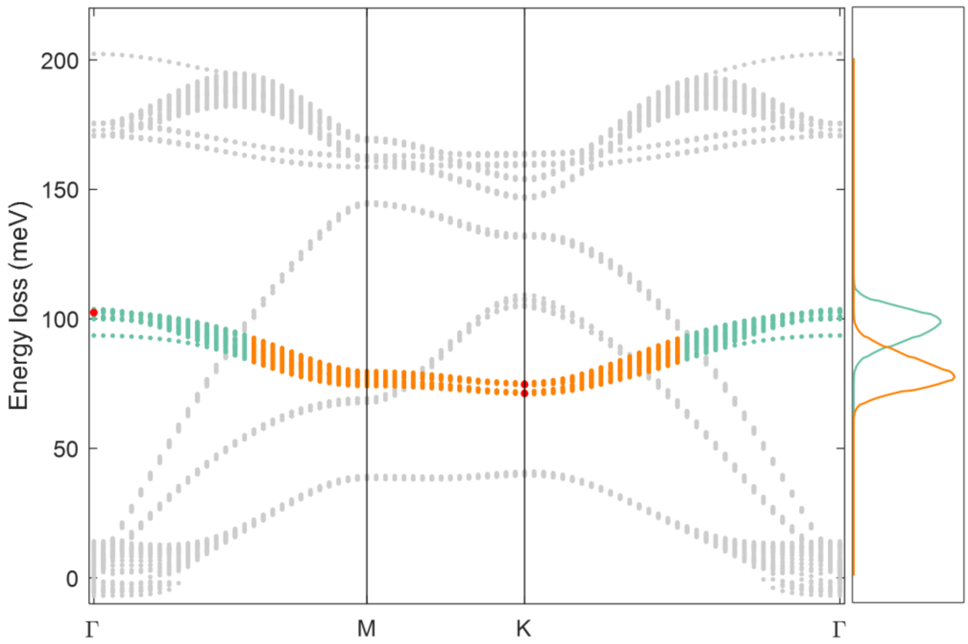

Supplementary figure 6 | Vibration modes of h-BN calculated by DFPT. In the left panel, each dot represents one phonon mode, while the modes highlighted by red color correspond to the typical Brillouin Zone center (q = Γ, ω = 102.468 meV) and Brillouin Zone boundary (q = K, ω = 71.201 meV & ω = 74.691 meV) out-of-plane optical phonon modes persented in Fig. 2c. Low q modes are labeled by green and high q modes are labeled by orange. The right panel shows the out-of-plane phonon DOS corresponding to low q and high q modes respectively.

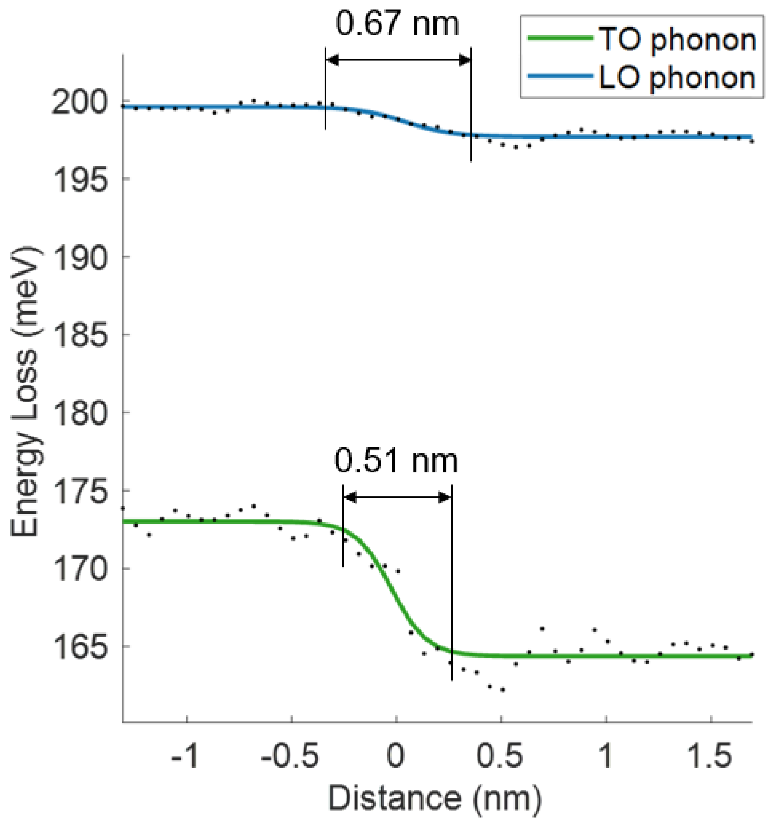

Supplementary figure 7 | The phonon transport of LO and TO modes in h-^{10}BN/h-^{11}BN. The black dots are fitted phonon energy of each spectrum, and the blue and green solid lines are fitting of black dots by the Logistic function, presenting the transition width of LO and TO is 0.67 nm and 0.51 nm respectively, much shorter than the width of ZO phonons.

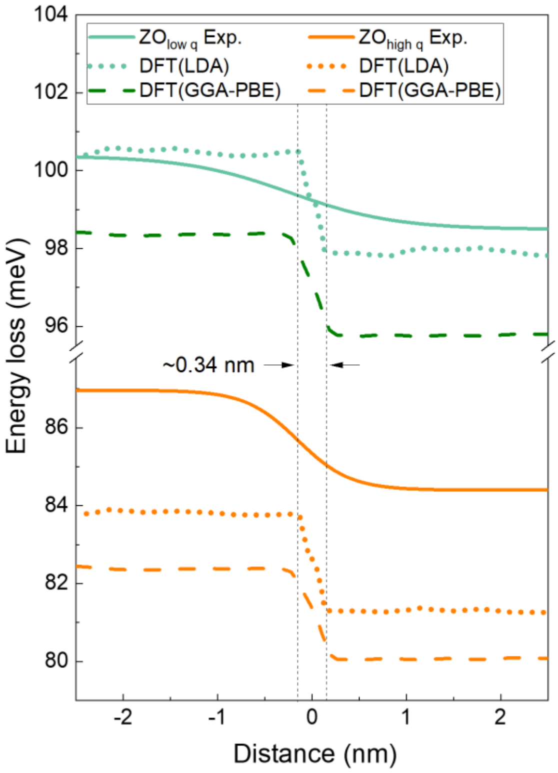

Supplementary figure 8 | Comparison of DFPT Calculations and the experimental ZO phonon transitions. The dotted cyan and orange lines are the energy change of ZO modes around the interface calculated by DFT with LDA functional, while the dashed ones are calculated by DFT with GGA-PBE functional. The solid lines are fitted from experimental results. Both DFT calculations show a very sharp transition at the interface, whose transition length is ~0.34 nm (one atomic layer), suggesting phonon transition at the isotopic interface of h-BN involves effects beyond harmonic approximation and Born-Oppenheimer approximation. It is worth mentioning that the phonon energy difference between the two calculations and experimental results is about 5 meV. Such a deviation is likely to be mainly caused by the limitation of accuracy of the exchange-correlation functional used. Improvement of DFT methods are needed to reach quantitative agreement with experiment (within meV) for phonon dispersion, but the two calculations suggest the width of the phonon transition layer is not affected by the choice of the exchange correlation functional.

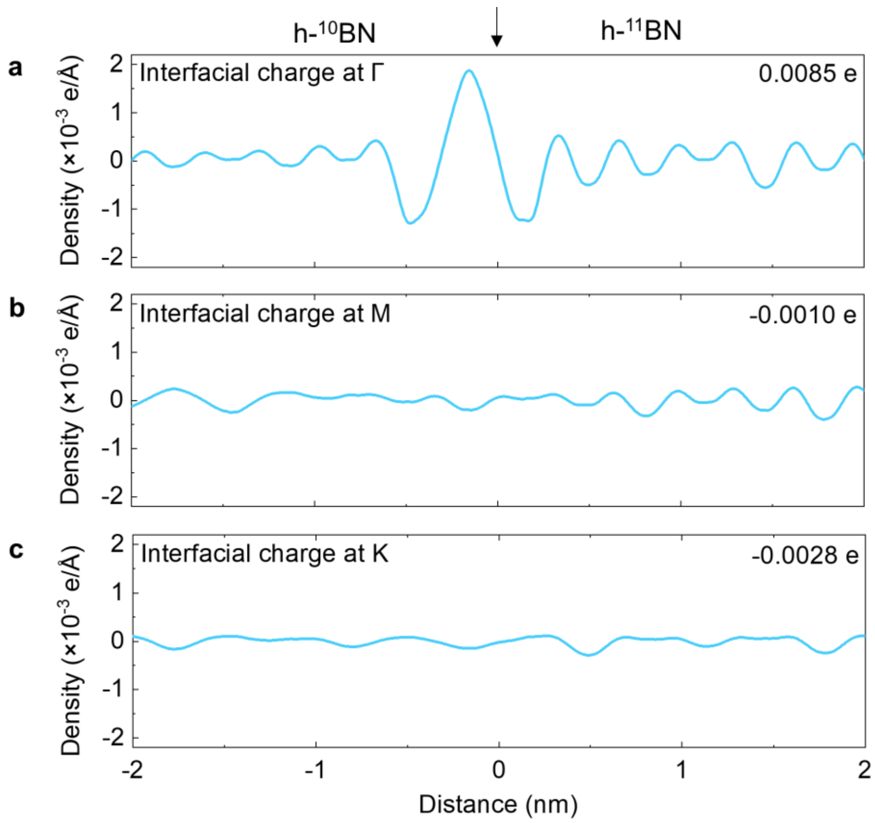

Supplementary figure 9 | Phonon induced differential charge density at h-^{10}BN/h-^{11}BN interface. The line profile of differential charge density induced by ZO phonon at **(a)** Γ, **(b)** M and **(c)** K point along the [0001] direction. Numbers at right-top corner of each panel show the total interfacial charge in the adjacent unit cell of the interface each side. As can be seen, interfacial charge induced by ZO phonon at Γ point is much higher than that at M/K point, indicating stronger interfacial electron-phonon coupling of $ZO_{low\ q}$.

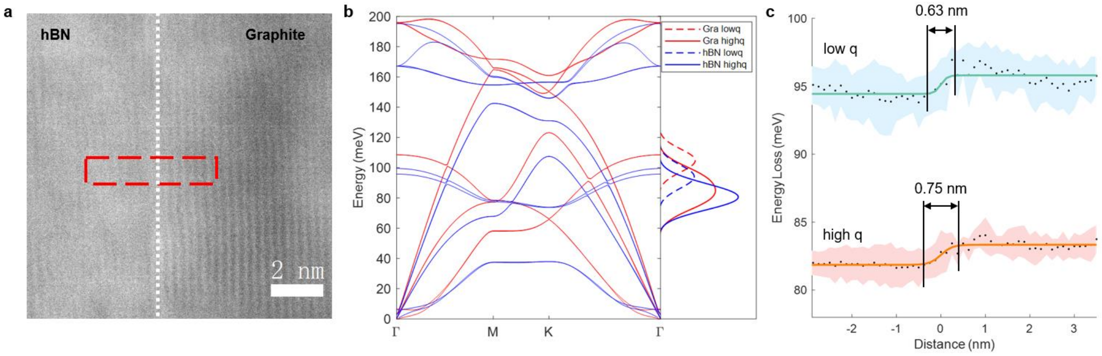

Supplementary figure 10 | The phonon transport of ZO modes in h-BN/graphite. **(a)** the HAADF image of h-BN/Graphite heterostructure. The white dashed line indicates the interface, and the red dashed rectangle labels the EELS acquisition region. **(b)** the phonon dispersion of graphite (red) and h-BN (blue), and the projected phonon density of states for graphite $ZO_{low\ q}$ (red dashed line), graphite $ZO_{high\ q}$ (red solid line), h-BN $ZO_{low\ q}$ (blue dashed line) and h-BN $ZO_{high\ q}$ (blue solid line). **(c)** Quantitative energy variation of the ZO modes in h-BN/graphite heterostrucutre. The black dots are fitted phonon energy of each spectrum, and the error bars are standard deviation shown as the orange and cyan shades. The orange and cyan solid lines are fitting of black dots by the Logistic function, presenting the transition width of $ZO_{low\ q}$ and $ZO_{high\ q}$ is 0.63 nm and 0.75 nm respectively. This is possibly due to the C atoms in graphite are neutral so the phonon induced dipole in hBN can hardly affect the vibration in graphite.

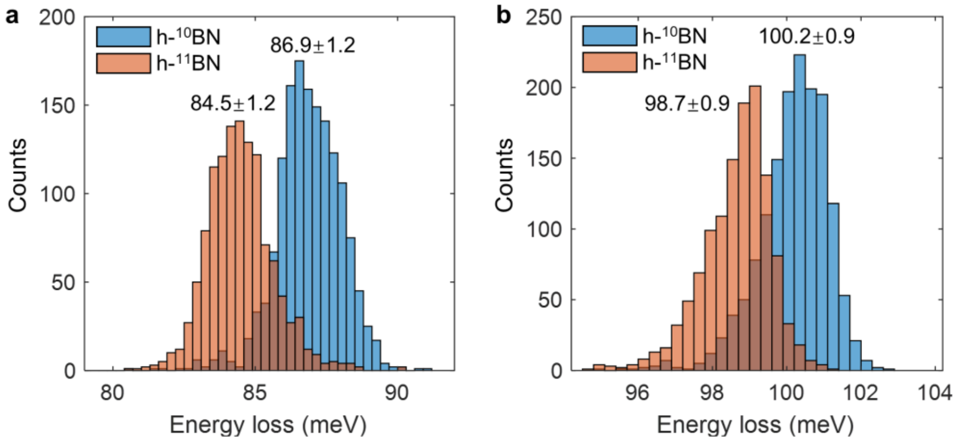

Supplementary figure 11 | Fitted energy distributions of $ZO_{high\ q}$ and $ZO_{low\ q}$. Histogram of fitted peak positions of **(a)** $ZO_{high\ q}$ and **(b)** $ZO_{low\ q}$ from 1500 acquisitions in h-^{10}BN/h-^{11}BN heterostructure, demonstrating clear separation of the isotopes.

Reference:

[1] P. Giannozzi, S. Baroni, N. Bonini, M. Calandra, R. Car, C. Cavazzoni *et al.* QUANTUM ESPRESSO: a modular and open-source software project for quantum simulations of materials. ***J Phys-Condens Mat*** 21, 395502 (2009).

[2] P. Giannozzi, O. Andreussi, T. Brumme, O. Bunau, M. Buongiorno Nardelli, M. Calandra *et al.* Advanced capabilities for materials modelling with Quantum ESPRESSO. ***J Phys-Condens Mat*** 29, 465901 (2017).

[3] D. Vanderbilt. Soft Self-Consistent Pseudopotentials in a Generalized Eigenvalue Formalism. ***Phys Rev B*** 41, 7892-7895 (1990).

[4] J. P. Perdew & A. Zunger. Self-Interaction Correction to Density-Functional Approximations for Many-Electron Systems. ***Phys Rev B*** 23, 5048-5079 (1981).

[5] J. P. Perdew, K. Burke & M. Ernzerhof. Generalized gradient approximation made simple. ***Phys Rev Lett*** 77, 3865 (1996).

[6] R. S. Qi, R. C. Shi, Y. H. Li, Y. W. Sun, M. Wu, N. Li *et al.* Measuring phonon dispersion at an interface. ***Nature*** 599, 399-403 (2021).

[7] S. Plimpton. Fast Parallel Algorithms for Short-Range Molecular-Dynamics. ***J Comput Phys*** 117, 1-19 (1995).

[8] A. Kinaci, J. B. Haskins, C. Sevik & T. Cagin. Thermal conductivity of BN-C nanostructures. ***Phys Rev B*** 86, 115410 (2012).

[9] Y. H. Li, M. Wu, R. S. Qi, N. Li, Y. W. Sun, C. L. Shi *et al.* Probing Lattice Vibrations at SiO_2/Si Surface and Interface with Nanometer Resolution. ***Chinese Phys Lett*** 36, 026801 (2019).

[10] K. Venkatraman, B. D. A. Levin, K. March, P. Rez & P. A. Crozier. Vibrational spectroscopy at atomic resolution with electron impact scattering. ***Nat Phys*** 15, 1237-1241 (2019).
